# A Comprehensive Transcriptomic Analysis of Arsenic-Induced Bladder Carcinogenesis

**DOI:** 10.3390/cells11152435

**Published:** 2022-08-05

**Authors:** Vaibhav Shukla, Balaji Chandrasekaran, Ashish Tyagi, Ajit Kumar Navin, Uttara Saran, Rosalyn M. Adam, Chendil Damodaran

**Affiliations:** 1 Rangel College of Pharmacy, Texas A&M University, College Station, TX 77843, USA; 2 Department of Urology, Boston Children’s Hospital, and Department of Surgery, Harvard Medical School, Boston, MA 02115, USA

**Keywords:** arsenic, bladder cancer, differential gene expression, stem cells, KEGG and GO enrichment

## Abstract

Arsenic (sodium arsenite: NaAsO2) is a potent carcinogen and a known risk factor for the onset of bladder carcinogenesis. The molecular mechanisms that govern arsenic-induced bladder carcinogenesis remain unclear. We used a physiological concentration of NaAsO2 (250 nM: 33 µg/L) for the malignant transformation of normal bladder epithelial cells (TRT-HU1), exposed for over 12 months. The increased proliferation and colony-forming abilities of arsenic-exposed cells were seen after arsenic exposure from 4 months onwards. Differential gene expression (DEG) analysis revealed that a total of 1558 and 1943 (padj < 0.05) genes were deregulated in 6-month and 12-month arsenic-exposed TRT-HU1 cells. The gene ontology (GO) and Kyoto Encyclopedia of Genes and Genomes (KEGG) analysis revealed that cell proliferation and survival pathways, such as the MAPK, PI3K/AKT, and Hippo signaling pathways, were significantly altered. Pathway analysis revealed that the enrichment of stem cell activators such as *ALDH1A1*, *HNF1b*, *MAL*, *NR1H4*, and *CDH1* (*p* < 0.001) was significantly induced during the transformation compared to respective vehicle controls. Further, these results were validated by qPCR analysis, which corroborated the transcriptomic analysis. Overall, the results suggested that stem cell activators may play a significant role in facilitating the arsenic-exposed cells to gain a survival advantage, enabling the healthy epithelial cells to reprogram into a cancer stem cell phenotype, leading to malignant transformation.

## 1. Introduction

Arsenic (As) is a naturally occurring metalloid classified as a group 1 carcinogen to humans by the International Agency for Research on Cancer (IARC). Chronic environmental exposure to inorganic arsenic (In-As) has been frequently associated with different types of cancer, which include squamous cell carcinoma of the skin [1,2] and lung [3,4], as well as bladder cancers [5,6]. The bladder is one of the identified target tissues for arsenic toxicity as some of the toxic methylated metabolites of In-As (thioarsenicals) excreted through urine have been detected in organs, particularly in the bladder in in vitro and in vivo model systems [7].

A clear dose-response relationship between As exposure and bladder cancer incidence was found in the population residing or working in As-polluted areas [8]. A concentration of 100–150 µg/L of In-As in drinking water would be sufficient to exert toxic effects, such as an increased risk of bladder cancer [9]. The National Research Council (NRC) has reported that lifetime exposure to As-contaminated drinking water (10 µg/L) would result in an elevated risk of bladder cancer [6]. The epigenetic modulation of tumor suppressor protein p53, Cyclin-dependent kinase inhibitor 2A (p16), Ras Association Domain Family Member 1 (*RASSF1A*), and the Serine Protease 3 (*PRSS3*) gene [10,11], as well as genetic changes, such as deletion, mutation, and aneuploidy [12,13], have been suggested as primary drivers of As-induced cytotoxicity. In addition, As-induced mitochondrial DNA mutation causes nuclear DNA damage and activates mitochondrial reactive oxygen species (ROS) that regulate cell proliferation [14]. Recent studies have also revealed that the normal stem cells responsible for self-renewal, differentiation, and cancer initiation may be the direct target of carcinogens. Chronic As exposure transforms normal stem cells into a cancer stem cell (CSC) phenotype in prostate and renal in vitro models [15,16,17].

Although the carcinogenic property of As has been established in different organs or cancer types, the precise molecular mechanism responsible for bladder carcinogenesis has not been fully elucidated. Most studies have shown the pleiotropic effects of arsenic after 4 to 6 months of exposure in cell culture models [15,17]. Thus, there is no suitable model to study the chronological molecular alterations during chronic As-exposure in bladder epithelial cells.

In the current study, to prepare the in vitro arsenic model, we have chronically exposed hTERT-immortalized bladder epithelial cells (TRT-HU1) to a low, physiologically relevant concentration of 250 nM sodium arsenite for over 12 months. These cells underwent phenotypic changes consistent with malignant transformation, i.e., increased proliferation and anchorage-independent growth. We have performed transcriptomic analysis of TRT-HU1 cells exposed to As for 6 months or 12 months to understand the complex molecular changes associated with malignant transformation. Gene set enrichment analysis (GSEA) indicated that upregulated genes are significantly enriched in cell proliferation, migration, and metastasis pathways. More precisely, stem cell regulatory pathways and genes such as Aldehyde Dehydrogenase 1 Family Member A1 (*ALDH1A1*), HNF1 Homeobox B (*HNF1B*), and Nuclear Receptor Subfamily 1 Group H Member 4 (*NR1H4*) were sequentially upregulated. These results demonstrate a strong association between transformative changes and gene expression patterns that can be further explored to identify and establish potential candidate biomarkers for the diagnosis, prognosis, and treatment of bladder cancer.

## 2. Materials and Methods

### 2.1. Cell Lines and Reagents

The hTERT-immortalized human urothelial cell line (TRT-HU1) was provided by Rosalyn M Adam (Boston Children’s Hospital and Harvard Medical School, Boston, MA 02115, USA) and checked for mycoplasma periodically. TRT-HU1 cells were maintained in Dulbecco’s Modified Eagle Medium (DMEM, GIBCO, US) containing 2 mM L-glutamine and 110 mg/L sodium pyruvate, supplemented with 10% fetal bovine serum and 1% antibiotic and antimycotic solution in a humified atmosphere of 5% CO_2_ at 37 °C. Briefly, for the transformation assays, 1.5 × 10^5^ TRT-HU1 cells were seeded in 100 mM dishes, and once the cells were attached, they were treated with either vehicle or 250 nM NaAsO_2_ (RICCA, Arlington, TX, USA). The media were changed weekly twice, and cells were split once a week. This step was repeated up to 12 months. Arsenic (1 mM) stock was prepared in distilled water and diluted to 250 nM during the treatment.

### 2.2. Cell Proliferation in Monolayer Culture

TRT-HU1 cells were exposed to sodium arsenite (250 nM) for up to 12months.Changes in cell proliferation weremeasured using trypan blue assay. Briefly, the cells were seeded at a density of 0.5 × 10^5^ cells/mL in complete medium. Following 48 h of cell growth, As (250 nM final concentration)or vehicle were added to cells. After 96 h, cells were trypsinized, washed, and resuspended in PBS containing 0.4% trypan blue. According to established protocol, a hemocytometer was used to count the number of viable cells.

### 2.3. Anchorage-Independent (Colony Formation) Growth Assay

TRT-HU1 cells exposed to sodium arsenite for 2, 4, 6, 8, 10, and 12 months were seeded at 1 × 10^4^ in 2 mL 0.35% agar in DMEM/FBS, overlaid on 2 mL of 0.7% agar in DMEM/FBS, in six-well plates. The cells were fed with fresh medium every 2–3 days. After 14 days of incubation, colonies were visualized by staining with crystal violet and images captured using an Evos XL microscope (Life Technologies, Carlsbad, CA, USA). Metabolically active colonies, having more than 10 cells, were counted as positive by two investigators (US and BC). All experiments were run in triplicate, and data are representative of two independent trials.

### 2.4. Spheroid Formation

Cell spheroids were produced utilizing the forced floating technique on 96-well ultra-low attachment plates (Corning, Corning, NY, USA). Each well was filled with a single-cell suspension of TRT-HU1 and transforming cells at a density of 1 × 10^3^ cells in 200 µL of respective culture media supplemented with Matrigel (Corning). Then, during a 7-day culture period in triplicate, the morphology of the spheroids and cell growth were examined.

### 2.5. Library Construction and RNA Sequencing

Total RNA was isolated from TRT-HU1 cells and transforming cells (6 and 12 months) using TRIzol (Thermo Fisher Scientific, Waltham, MA, USA). The quantity and quality of RNA were assessed using aNano Photometer spectrophotometer (Fisher Scientific). The RNA was stored at −80 °Cuntil further use. In accordance with the manufacturer’s instructions, the cDNA library was prepared using Novogene Bioinformatics Technologies (Beijing, China) and a NEBNext Ultra TM RNA library kit for Illumina (New England Biolabs, Ipswich, MA, USA) by Novogene Bioinformatics Technologies Co. Ltd. Briefly, mRNA was isolated from RNA samples using poly-T oligo attached magnetic beads, fragmented to a size of 200 bp with divalent cations at high temperature, and then reverse-transcribed into cDNA using random hexamer primers and MMuLV Reverse transcriptase. RNase H, dNTP, and DNA polymerase I were used to making the second strand of cDNA, followed bythe amplification of double-stranded cDNA using Phusion high-fidelity DNA polymerase, universal primers, and the index (X) primer. The amplified cDNA library was then purified using the AMPure XP system (Beckman Coulter, Brea, CA, USA), and the library quality was evaluated using the Agilent bioanalyzer 2100 system. The enriched product was sequenced using the Illumina Hiseq 2000/2500 platform, and paired ends were generated.

### 2.6. Computational Data Analysis

FASTP was used to process the raw files, and Q20, Q30, and GC content were calculated [18]. Spliced transcripts were aligned to a reference (STAR) software for high-quality mapping reads to the reference genome [19]. The read counts for each gene were calculated using Feature Counts [20]. Based on the gene length and mapped reads, RPKM (reads per kilobase of the transcript, per million, and mapped reads) was calculated using the previously described method [21]. The differential expression analysis between 0-month, 6-month, and 12-month samples was carried out using DeSeq2 with biological replicates [22]. logFC (log2 fold change) >2 and <−2 were assigned as up- and downregulated mRNAs with P_adj_ (adjusted *p* value) <0.05.

### 2.7. Reverse Transcription and Real-Time PCR Quantitation

cDNA was synthesized from total RNA using iScript Reverse Transcription Supermix (BioRad, Hercules, CA, USA) following the manufacturer’sprotocol. The expressionof genes was determined using Sso Advanced Universal SYBR GreenSupermix (BioRad, Hercules, CA, USA). β-actin was used as an endogenous control. Real-time PCR was performed using the CFX-connect real-time system (BioRad, Hercules, CA, USA). Primer sequencesare provided in Supplementary Table S1. All experiments were performed in triplicate.

### 2.8. Functional Enrichment Analysis

Heatmap and principal component analysis (PCA) were performed using ClustVis [23]. A volcano plot was generated using VolcaNoseR [24]. Gene set enrichment analysis (GSEA) was done using GSEA software version 4.2.2 [25]. ShinyGO [26] was used to predict the Kyoto Encyclopedia of Genes and Genomes (KEGG) pathways. Gene ontology (GO) analysis, including biological process (BP), cellular component (CC), and molecular function (MF), was performed by PANTHER [27] and SRplot (https://www.bioinformatics.com.cn/en (accessed on: 31 June 2022)) [28]. The association of gene expression profiles with pathway activity specific to bladder cancer was performed using GSCALite [29].

### 2.9. Statistical Analysis

The GraphPad Prism (GraphPad Software, Inc., La Jolla, CA, USA) was used forstatistical analysis. Two-way ANOVA and student’s *t*-test were used to the analyze the difference between treatment groups. The data are presented as the mean ± SD. The significance of the differences between the groups was determined using the unpaired student’s *t*-test, and multiple comparisons between groups were performed using a one-way ANOVA with a post hoc Dunnett’stest. *p* < 0.05 was considered significant.

## 3. Results

### 3.1. Chronic Exposure to As Induces Malignant Transformation of TRT-HU1 Cells

**Figure 1:**
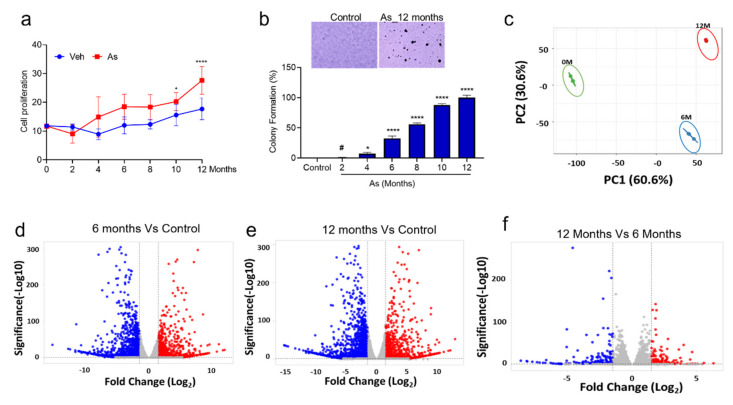
Determination of cell viability, colony formation ability of transforming cells, and identification of differentially expressed genes from RNA seq datasets. (**a**) The cell viability was measured by the intake of trypan blue by dead cells in control and transforming cells (0, 2, 4, 6, 8, 10, and 12 months). (**b**) Colony-forming assays on 0, 2, 4, 6, 8, 10, and 12 months transforming TRT-HU1 cells were performed. Colonies were manually counted after being stained with crystal violet. All experiments were performed in triplicate. One-way ANOVA with multiple comparison tests was used to calculate the statistical significance between different experimental groups *, *p* < 0.05; and ****, *p* < 0.0001, # Not significant (**c**) PCA plot analysis of differentially expressed genes in three datasets. The volcano plot analysis of differentially expressed genes between (**d**) 6-month against 0-month, (**e**) 12-month against 0-month, and (**f**) 12-month against 6-month is plotted on the X axis, and false discovery rate (FDR) significance is plotted on the Y axis (-log10 scale). The grey dots represent no significant change, red dots represent logFC of >2 and FDR < 0.05, and blue dots represent logFC < −2 and FDR < 0.05.

To understand the effect of chronic As exposure on TRT-HU1 cells, we performed cell viability assays periodically in triplicates for up to 12 months. Initially, As exposure causes toxicity to healthy bladder epithelial cells (up to 3 months); hence, a reduction in the viability was observed. However, from the fourth month onwards, the proliferation was significantly increased in As-exposed cells compared to vehicle-treated TRT-HU1 (Figure 1a). Next, we performed anchorage-independent growth assays to determine the malignant phenotype of As-exposed cells. The colonies began forming after four months, and a time-dependent, significant increase in the number of colonies was observed in As-exposed cells compared to vehicle-treated TRT-HU1 cells (Figure 1b, *p* < 0.0001). A more than a 20-fold increase in the number and size of colonies in As-exposed cells (12th month) was evident compared with their respective controls.

### 3.2. Identification of Differentially Expressed Genes (DEGs) in As-Induced Transformation

It is evident from the cell proliferation and colony formation assays that TRT-HU1 cells are transforming into malignant phenotypes following chronic exposure to As. To decipher the molecular events associated with these changes, we performed a global transcriptomic analysis on vehicle and As-exposed (6- and 12-month) TRT-HU1 cells. Principal components analysis (PCA) suggested distinct mRNA expression patterns in the vehicle and As-exposed cells (Figure 1c). Therefore, we performed a volcano plot-based filtering analysis to identify genes with significantly differential expression among these groups. A total of 770 genes were significantly upregulated (padj ≤ 0.05), while 1173 genes were downregulated (padj ≤ 0.05) in cells at 12 months (12M) as compared to vehicle-treated TRT-HU1 cells (Figure 1e). Similarly, we identified 584 upregulated (padj ≤ 0.05) and 974 downregulated genes (padj ≤ 0.05) in cells at 6 months (6M) as compared to vehicle-treated cells (Figure 1d). Interestingly, the number of genes differentially expressed at 12M compared to 6M was drastically reduced. There were 126 genes (padj ≤ 0.05) upregulated and 155 downregulated (padj ≤ 0.05) at 12M compared to 6M (Figure 1f). These results indicate that although both 6-month and 12-month populations have a high number of differentially expressed genes, fewer than expected genes might play a critical functional role in the complete transformation of TRT-HU1 cells. We identified 468 genes that were upregulated in both 6 and 12 months of arsenic treatment; however, 126 genes appear to be specific to 12 months compared to other treatment groups (6 months of arsenic treatment). Our results suggest that 28 genes were responsible for the chronological progression of arsenic-induced carcinogenesis (Supplementary Figure S5). Similarly, for downregulated genes, we identified 51 genes common in all treatment groups (Supplementary Figure S6). Next, we performed a functional analysis of these differentially expressed genes to gain a comprehensive insight into the changes that may be responsible for malignant transformation.

### 3.3. In Silico Functional Analysis of DEGs

**Figure 2:**
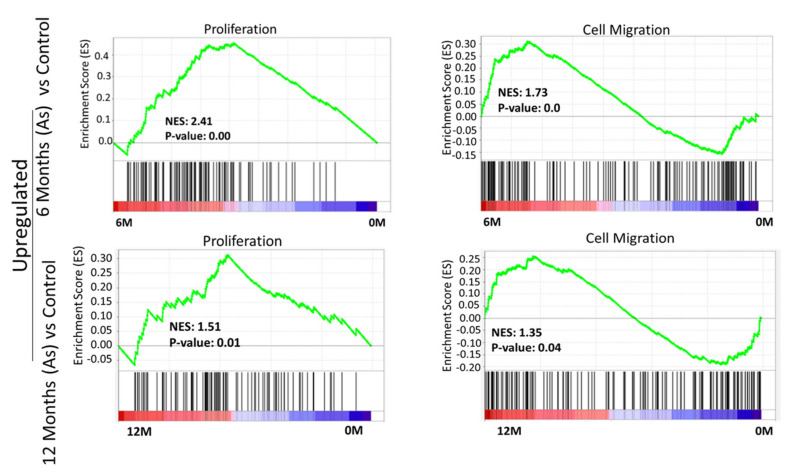
Gene set enrichment analysis (GSEA) of 6-month and 12-month As-exposed cells compared to control with positive enrichment score. GSEA curves of the list of differentially expressed genes in 6-month vs. 0-month and 12-month vs. 0-month. Gene set enrichment at the top of the ranked list is indicated by a positive enrichment score (ES).

To validate the molecular changes, we performed gene set enrichment analysis (GSEA) of the whole genome at the transcription level. GSEA of As-exposed cells at 6 and 12 months revealed the significant enrichment of genes responsible for the initiation and progression of bladder cancer, including those involved in migration and proliferation (Figure 2), in agreement with our phenotypic assays (Figure 1b).

Interestingly, we observed a negative association of autophagy, tumor suppressor 53 (TP53), and tumor suppressor 63 (TP63) target genes in As-exposed cells at 6M and 12M (Supplementary Figure S1). The cellular transformation signatures were enriched in As-treated cells exposed for 6 months (Supplementary Figure S2), suggesting transformation initiated at this time point.

### 3.4. GSEA Confirmed Functional Changes at the Transcripts Level during Transformation in the As Exposed Cells

To understand which DEGs are actively involved or inhibited in different biological pathways, we mapped our gene signatures onto KEGG pathways. In total, there were 101 pathways significantly associated with DEGs in As-exposed cells at 12 months compared with vehicle-treated cells (FDR < 0.05). Similarly, a lesser enrichment (67 pathways: FDR < 0.05) was seen in cells exposed to As for 6 months compared to control cells. A total of 65 common pathways were significantly deregulated in 6 and 12 months As-exposed cells relative to their respective controls (FDR < 0.05), including PI3K-Akt, TGF-beta, Wnt, MAPK, Hippo, and notch signaling pathways (Supplementary Table S2). Among these, the pluripotency of stem cell pathways was more prominent in the As-exposed cells than the vehicle-treated cells. Further, the PANTHER tool was used to perform GO in terms of biological processes (BP), molecular function (MF), and cellular component (CC).

**Figure 3:**
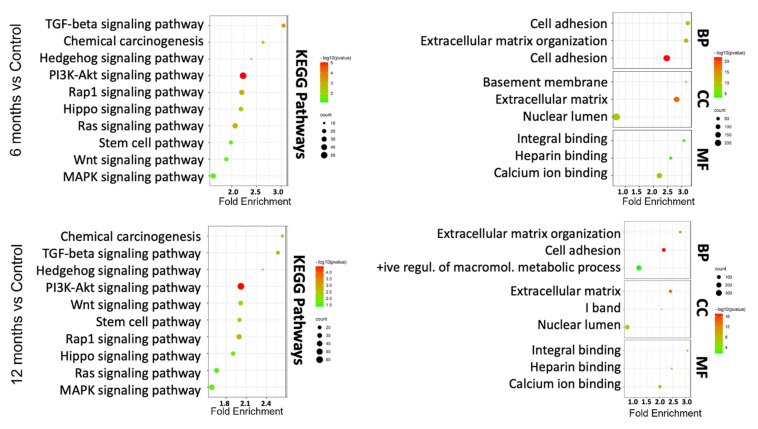
KEGG pathway and GO term analysis of differentially expressed genes based on RNA-seq data for 6-month and 12-month As-exposed cells compared to control cells. Each bubble’s color and size correspond to the amount of differentially expressed mRNAs enriched in GO or KEGG pathway, respectively. The cut-off for choosing to GO and KEGG keywords was *p* < 0.05.

The results demonstrated that DEGs were mainly involved in extracellular matrix organization, cell adhesion, and the positive regulation of macromolecule metabolic process in the BP category; in the extracellular matrix, I band, and nuclear lumen in the CC category; and integrin binding, heparin-binding, and calcium ion binding in the MF category (Figure 3). Overall, KEGG pathway analysis and GO terms are shown in Supplementary Figures S3 and S4. These GO terms have been associated with bladder cancer studies [30,31]. These analyses indicate that TRT-HU1 cells acquired stem-cell characteristics and cancer hallmarks during transformation following As exposure.

### 3.5. Validation of Transcriptome Results by qRT-PCR

**Figure 4:**
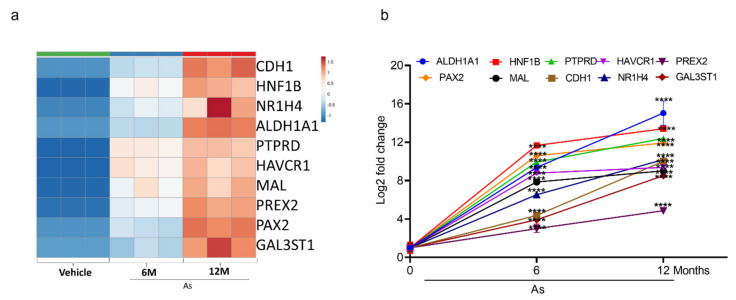
Expression validation of selected genes (RNA-seq datasets) using qRT-PCR. Heatmap analysis showing the expression level of top 10 upregulated genes (**a**) in RNA-seq datasets. The expression for upregulated genes (**b**) was determined by quantitative RT-PCR in successive stages of transforming cells (0, 6, and 12 months). Expression levels were normalized to β-actin. Error bars indicate the standard deviation of triplicates. One-way ANOVA was used to calculate the statistical significance between vehicle control and treatment at each time point. (**** *p* < 0.0001).

**Figure 5:**
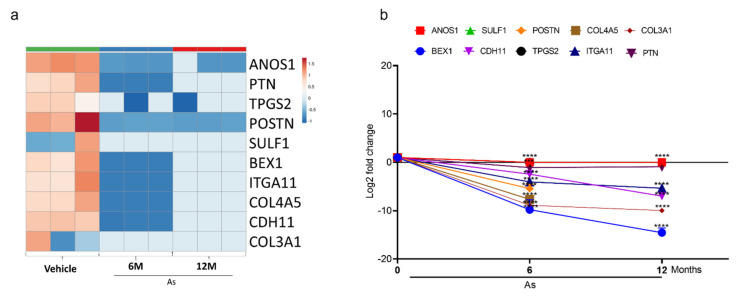
Expression validation of selected genes (RNA-seq datasets) using qRT-PCR. Heatmap analysis showing the expression level of top 10 downregulated genes (**a**) in RNA-seq datasets. The expression for downregulated genes (**b**) was determined by quantitative RT-PCR in successive stages of transforming cells (0, 6, and 12 months). Expression levels were normalized to β-actin. Error bars indicate the standard deviation of triplicates. One-way ANOVA was used to calculate the statistical significance between vehicle control and treatment at each time point. * *p* < 0.05, *** *p* < 0.001, and **** *p* < 0.0001.

To validate the top-10 upregulated (Figure 4a) and downregulated genes (Figure 5a from our DEGs analysis, we performed qRT-PCR analysis. We confirmed the mRNA expression of the 10 most upregulated genes (Phosphatidylinositol-3,4,5-Trisphosphate Dependent Rac Exchange Factor 2 (*PREX2*), Protein Tyrosine Phosphatase Receptor Type D (*PTPRD*), Hepatitis A Virus Cellular Receptor 1(*HAVCR1*), *HNF1B*, Mal, T Cell Differentiation Protein (*MAL*), *NR1H4*, Galac-tose-3-O-Sulfotransferase 1(*GAL3ST1*), Cadherin 1 (*CDH1*), *ALDH1A1*, and Paired Box 2 (*PAX2*) and most downregulated genes (Collagen Type III Alpha 1 Chain (*COL3A1*), Sulfatase 1(*SULF1*), Tubulin Polyglutamylase Complex Subunit 2 (*TPGS2*), Cadherin 11 (*CDH11*), Pleiotrophin (*PTN*), Collagen Type IV Alpha 5 Chain (COL4A5), Brain Expressed X-Linked 1 (*BEX1*), Integrin Subunit Alpha 11 (*ITGA11*), Anosmin 1 (*ANOS1*), and Periostin (*POSTN*)). The change in expression was measured as a Log2-fold change. *ALDH1A1* (15-fold) is the most upregulated gene, followed by *HNF1B* (13-fold), *PTPRD* (12-fold), *PAX2* (11-fold), *HAVCR1* (9-fold), and *NR1H4* (10-fold) in As-transformed cells compared to their respective controls (Figure 4b).

Similarly, a significant downregulation (Log2-fold) of *CDH11* (6-fold), *BEX1* (14-fold), and *ITGA11* (5-fold) was seen in As-transformed cells compared to vehicle-treated cells (Figure 5b). However, *ANOS1, SULF1, POSTN, TPGS2, COL3A1*, and *COL3A5* showed no expression in 12-month As-exposed cells. The gene set cancer analysis (GSCALite) was utilized to correlate the gene expression with bladder cancer (Table 1). The results demonstrated that most downregulated gene clusters are responsible for apoptotic signaling or PI3K/Akt pathway inhibition. However, upregulated genes were shown to activate cancer stem cells, EMT, hormone receptors, and tyrosine kinases, suggesting the alterations in the expression of these genes may lead to the transformation of healthy bladder epithelial cells.

### 3.6. Characterization of Stem Cells Properties

**Figure 6:**
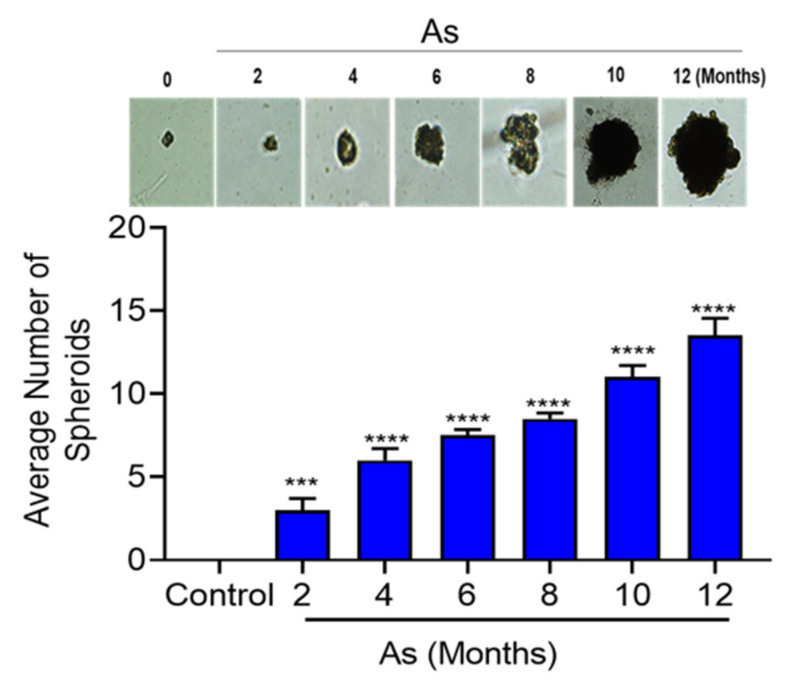
Sphere formation ability of transforming TRT-HU1 cells (0, 2, 4, 6, 8, 10, and 12 months). Control and transforming (2 × 10^3^ cells) were plated in 12-well ultra-low attachment plates, and allowed for 14 days, then the total number of spheres per well were counted. The horizontal bars represent the mean number of spheres, *** *p* < 0.001, and **** *p* < 0.0001.

Tumor-initiating stem cells, also known as cancer stem cells (CSCs), may allow cells to differentiate into multiple tumor cell types. Our in silico functional analysis showed that the pluripotency of stem cell signaling pathways was highly enriched, and those results were validated by qRT-PCR. Hence, we performed sphere formation assays on vehicle and As-exposed bladder epithelial cells. Figure 6 shows a gradual increase in the sphere formation efficiency from the fourth month onwards, and significant increases in both the number (13-fold) and the size of spheres were seen in cells at the 12th month of As-exposure.

## 4. Discussion

In this study, we demonstrate that chronic exposure to low concentrations of arsenic (sodium arsenate) transformed immortalized bladder epithelial cells (TRT-HU1) into malignant phenotypes. A systematic transcriptomic analysis revealed that an enrichment of the stem cell regulatory pathway might be responsible for the transformation. The induction of several stem cell regulators (*ALDH1A*, Sonic hedgehog signaling molecule (*SHH)*, *HNF1b*, *MAL*, *NR1H4*, and *CDH1*) in As-transformed cells appears to play a significant role in the malignant transformation of bladder epithelial cells. Interestingly, these stem cell regulators are expressed in bladder cancer specimens, suggesting these findings could be correlated with clinical parameters [32,33].

The acquisition of a stem cell-like phenotype has been proposed as a possible mechanism for the As-induced oncogenic transformation of prostate epithelial cells (RWPE-1). Exposure to inorganic As for up to 18 weeks at a concentration of 5 µM suppressed phosphatase and tensin homolog (*PTEN)* and increased the expression of self-renewal genes, such as *SHH* and POU class5 homeobox (*OCT-4*) [15]. Similarly, an increase in expression of *ALDH1A1* and *Oct-4* played a critical role in the As-induced transformation of human bronchial epithelial (HBE) cells [34]. Moreover, *ALDH1A1* was one of the few genes necessary for the As-mediated maintenance of stem cells. In our study, the induction of stem cell regulators such as *ALDH1A1* (15 fold), *HNF1b* (13 fold), *MAL* (9fold), *NR1H4* (10 fold), and *CDH11* [35,36,37,38,39] during the transformation of bladder epithelial cells suggests their possible involvement in the malignant transformation of TRT-HU1 cells. Further, in silico analysis suggests that stem cell regulation might have a strong survival selection advantage towards transformation.

Spheroid formation demonstrates the capacity of cells for self-renewal and for the initiation of tumors [40], which are characteristics of stem cells. RWPE-1 and HBE cells acquired stem-cell-like properties during arsenite-treatment, which led to spheroid formation and the transformation of both cell types [15,34]. We also observed a significant increase in spheroid formation during the transformation of TRT-HU1 cells compared to the respective matching control. These findings suggest that As reprograms healthy epithelial cells into a cancer stem cell phenotype as early as 4 months after exposure, eventually leading to malignant transformation.

Previous studies on As-induced bladder carcinogenesis were conducted in the immortalized urothelial cell line, UROtsa [41]. However, the possible contamination of UROtsa cells with T24 cells was reported subsequently, clouding the interpretation of prior findings [42]. Prior studies also used monomethylarsonous acid, MMA (III), which at a 50 nM dose for 12 weeks could be more toxic than sodium arsenate [43]. Here, we used a physiological concentration of arsenic (250 nM: 33 µg/L, the mean concentration of arsenic detected in BCa patients) to treat hTERT immortalized TRT-HU1 cells. Hence, the current study may be considered a better in vitro model to study the effect of As-induced transformation of bladder epithelial cells.

Another significant observation from the GSEA of the differential expressed genes (DEGs) is the correlation between DEGs from As-exposed TRT-HU1 cells with gene sets linked to malignant cell proliferation. We observed a more than three-fold increase in the expression of genes encoding proliferation markers (meiotic nuclear divisions 1 (*MND1*), thymosin beta 10 (*TMSB10*), cystatin B (*CSTB*), fibroblast growth factor 18 (*FGF18*), fibroblast growth factor receptor 1 *(FGFR1*), and fibroblast growth factor receptor 4 *(FGFR4*)) in As-exposed TRT-HU1 cells. Earlier studies have also shown an increase of *FGF18* and its receptors (*FGFR1* and *FGFR4*) responsible for increased proliferation in As transformed UROtsa cells [41]. In alignment with our GSEA observations, the KEGG analysis also revealed pathways associated with PI3K/Akt signaling, MAPK signaling, and Hippo signaling in As-transformed cells, which correlated with clinical findings in bladder cancer specimens [44,45,46]. Exposure to 2.5 µM of As for 24 weeks altered the signaling networks of p21, Akt, and MAPK and disrupted PPARα/δ-mediated lipid homeostasis in bronchial epithelial cell lines (BEAS-2B) [47]. Similarly, the acute exposure to arsenite has been found to activate PI3K and AKT signaling, which regulates hypoxia inducible factor 1 subunit alpha (*HIF-1*) and vascular endothelial growth factor (*VEGF*) expression by generating reactive oxygen species (ROS) in prostate cancer cells [48]. Our study also showed that the PI3K/AKT signaling and MAPK signaling pathways were significantly altered (FDR < 0.05). Nonetheless, whole-genome expression profiling and in silico pathway analysis have demonstrated that the chronic in vitro arsenic exposure of normal bladder epithelial cells (TRT-HU1) enhanced the proliferation and may be a predisposing factor in the malignant transformation of cells.

Apart from the prominent role of proliferation-associated pathways, another interesting observation from our study is the inhibition of genes/pathways associated with autophagy in As-exposed TRT-HU1 cells. Previous findings have suggested that acute As exposure at a concentration of 1 µM blocks autophagic flux, which results in the accumulation of p62 [49], and that autophagy is a self-protective mechanism against the arsenic-induced transformation of BEAS-2B cells [50]. In this respect, the inhibition of autophagy may have also contributed to the As-induced transformation of normal cells.

Our volcano plot and GO analysis showed the significant downregulation of tumor-suppressive genes, such as *ANOS1*, *CDH11*, *COL4A5*, *POSTN*, *PTN*, and *BEX1* in As- transformed cells, which is considered an early event of carcinogenesis. Furthermore, these changes were correlated with clinical specimens from different cancer types, including bladder cancer [51,52,53,54,55,56].

In summary, our study has shown that both pro-and anti-cancer signaling is activated after As exposure of non-transformed bladder epithelial cells, resulting in the dysregulation of cellular homeostasis. Furthermore, we have established an in vitro model using a physiological concentration of As in immortalized bladder epithelial cells for As-induced malignant transformation. Interestingly, we have also found that the expression of genes and signaling pathways affected by As exposure is interlinked. Given the significance of the genes and pathways identified in our study and other previous publications, future studies must be conducted to explore the involvement of these genes and pathways in the pathophysiology of bladder cancer.

## Figures and Tables

**Table 1:** Effect of mRNA expression on pathway activity based on TCGA-BLCA datasets.

Gene Symbol	Pathway	FDR	Class
Upregulated Genes			
ALDH1A1	EMT	9.75 × 10^−9^	Activation
CDH1	RTK	0.006827	Activation
GAL3ST1	PI3K/AKT	0.047569	Activation
HNF1B	RTK	0.000231	Activation
PTPRD	EMT	1.77 × 10^−15^	Activation
MAL	Hormone AR	0.000131	Activation
NR1H4	Hormone AR	8.8 × 10^−5^	Activation
PREX2	Hormone AR	0.01951	Activation
HAVCR1	Hormone AR	0.000703	Activation
Downregulated Genes			
SULF1	Apoptosis	0.006361	Activation
ANOS1	Hormone AR	0.002647	Inhibition
CDH11	Hormone AR	0.000904	Inhibition
COL3A1	Hormone AR	0.000184	Inhibition
COL4A5	Hormone ER	1.69 × 10^−7^	Inhibition
ITGA11	PI3K/AKT	0.018696	Inhibition
ITGA11	Hormone AR	0.006147	Inhibition
POSTN	Hormone AR	2.13 × 10^−6^	Inhibition

The differentially expressed genes’ (top 10 up- and downregulated genes) correlation with pathway activity (activation and inhibition).

## Data Availability

The datasets used and/or analyzed during the current study are available from the corresponding author on reasonable request.

## Supplementary Materials

The following supporting information can be downloaded at: https://www.mdpi.com/article/10.3390/cells11152435/s1, Figure S1: Gene set enrichment analysis (GSEA) of 6-month and 12-month As exposed cells compared to control; Figure S2: Gene set enrichment analysis (GSEA) of 12-month As exposed cells compared to 6-month. Figure S3: Overall, KEGG pathway and GO term analysis of differentially expressed genes based on RNA-seq data for 6 months As exposed cells compared to control cells. Figure S4: Overall KEGG pathway and GO term analysis of differentially expressed genes based on RNA-seq data for 12 months As exposed cells compared to control cells; Figure S5: Common upregulated genes between 12M vs 0M, 6M vs 0M and 12M vs 6M. Figure S6: Common downregulated genes between 12M vs 0M, 6M vs 0M and 12M vs 6M; Table S1: List of primers used in the study; Table S2: Common and unique pathways between 12M vs 0M and 6M vs 0M.
