# Supplementary material for: A Comprehensive Transcriptomic Analysis of Arsenic-Induced Bladder Carcinogenesis
Source: Cells. 2022 Aug 5;11(15):2435. doi: 10.3390/cells11152435 (PMC9367831; doi:10.3390/cells11152435)
Supplement: Supplementary file 1 [file cells-11-02435-s001.zip › cells-1852897-supplementary.pdf]

(a)

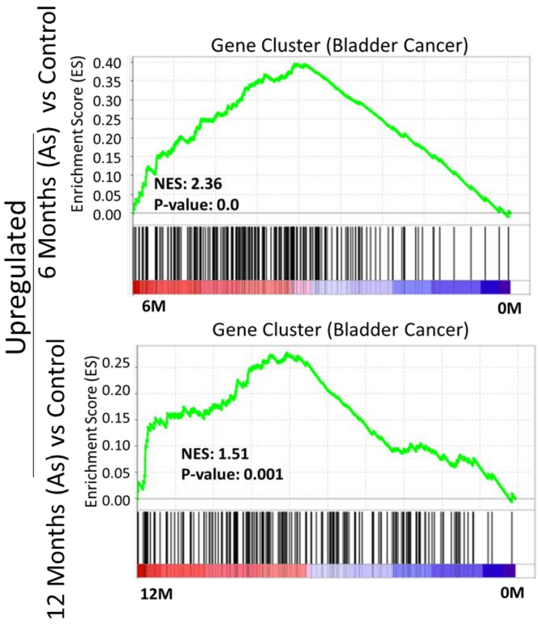

(b)

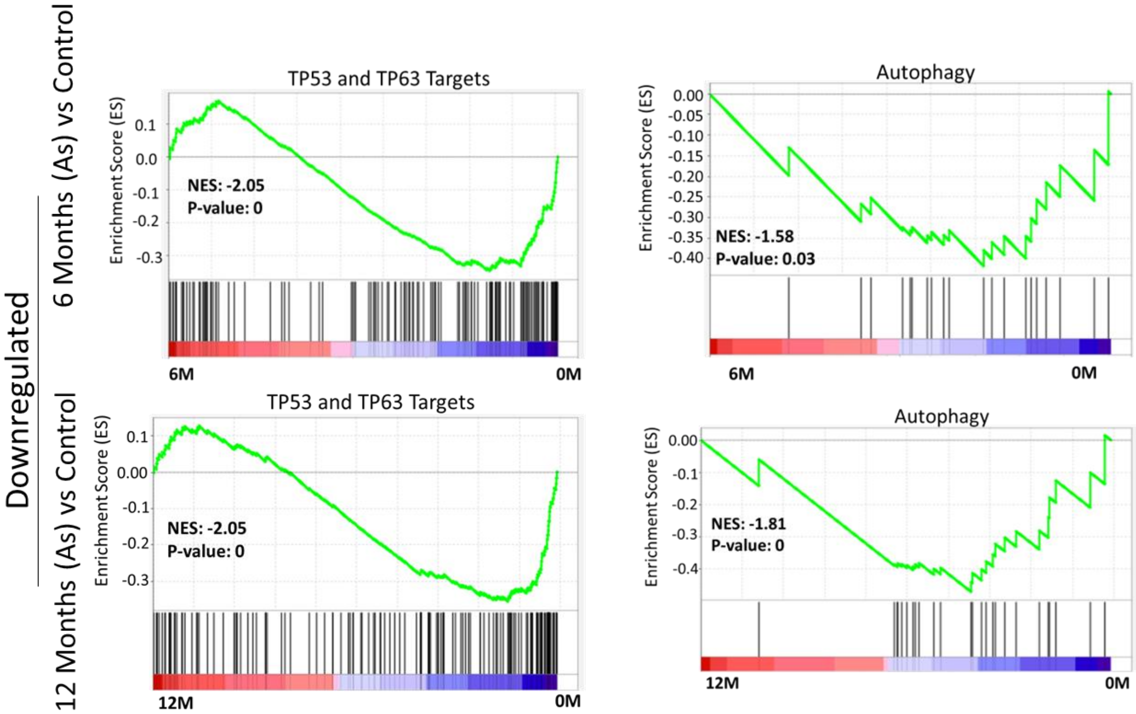

Supplementary Figure S1. Gene set enrichment analysis (GSEA) of 6-month and 12-month As exposed cells compared to control. **(a)** Gene set enrichment at the top of the ranked list is indicated by a positive enrichment score (ES). (**b)** Gene set enrichment at the bottom of the ranked list is indicated by a negative enrichment score (ES).

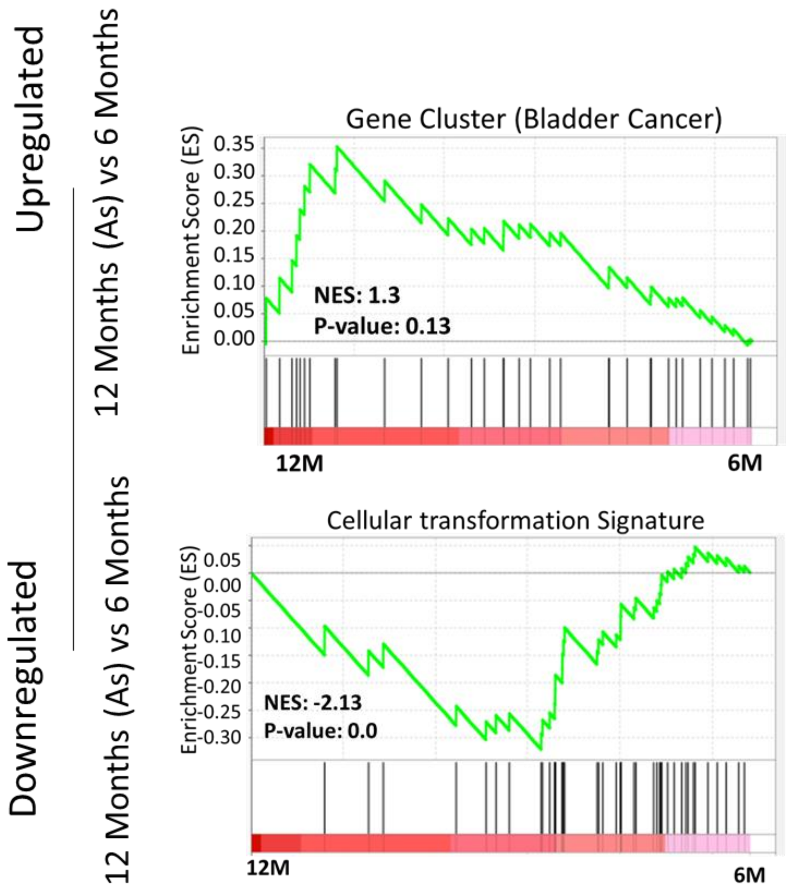

Supplementary Figure S2. Gene set enrichment analysis (GSEA) of 12-month As exposed cells compared to 6-month. Gene set enrichment at the top of the ranked list is indicated by a positive enrichment score (ES). Gene set enrichment at the bottom of the ranked list is indicated by a negative enrichment score (ES).

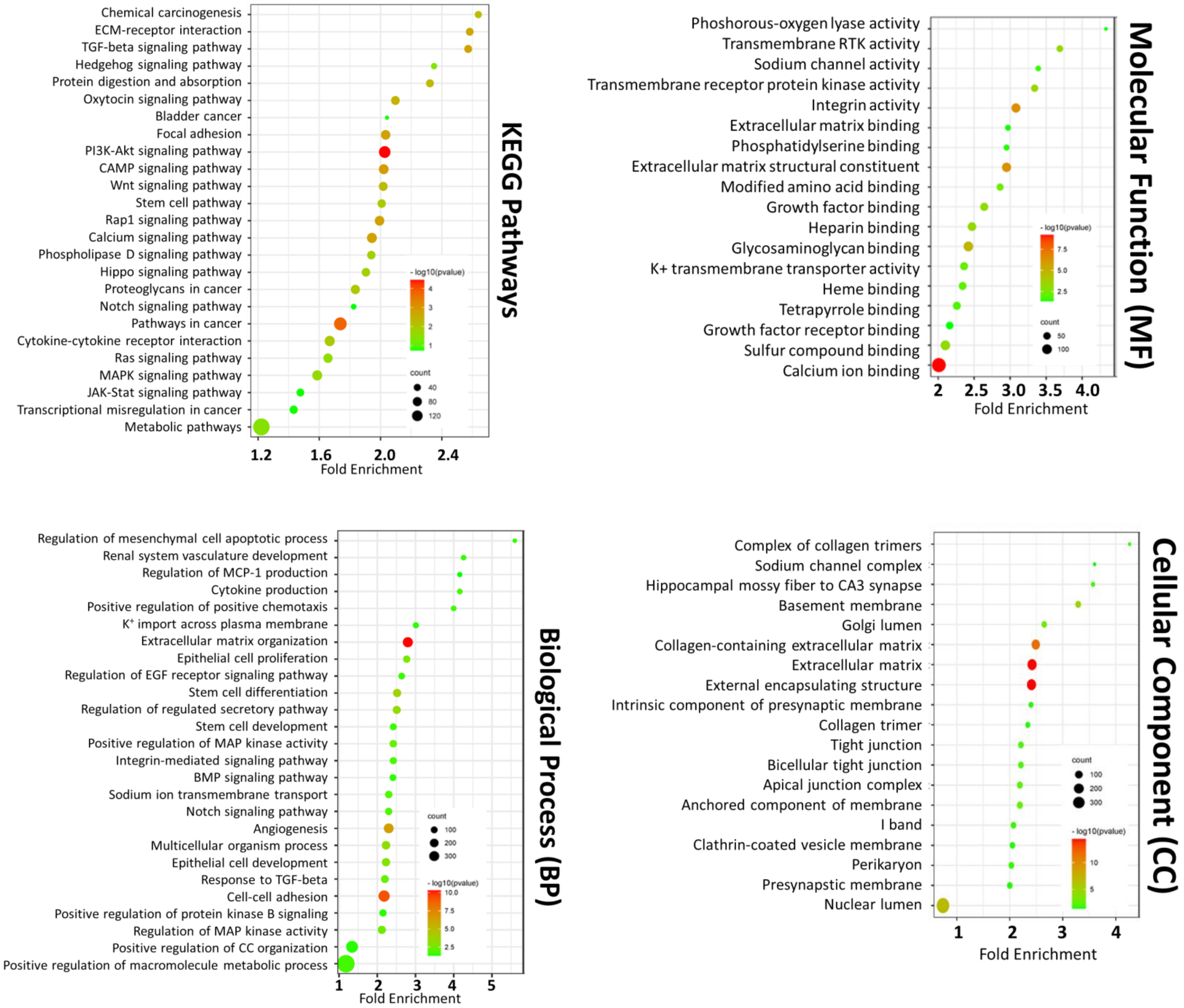

Supplementary Figure S3. Overall KEGG pathway and GO term analysis of differentially expressed genes based on RNA-seq data for 6 months As exposed cells compared to control cells. Each bubble's color and size correspond to the amount of differentially expressed mRNAs that are enriched in a certain GO keyword or KEGG pathway, respectively. The cut-off utilized to choose to GO and KEGG keywords were $p<0.05$.

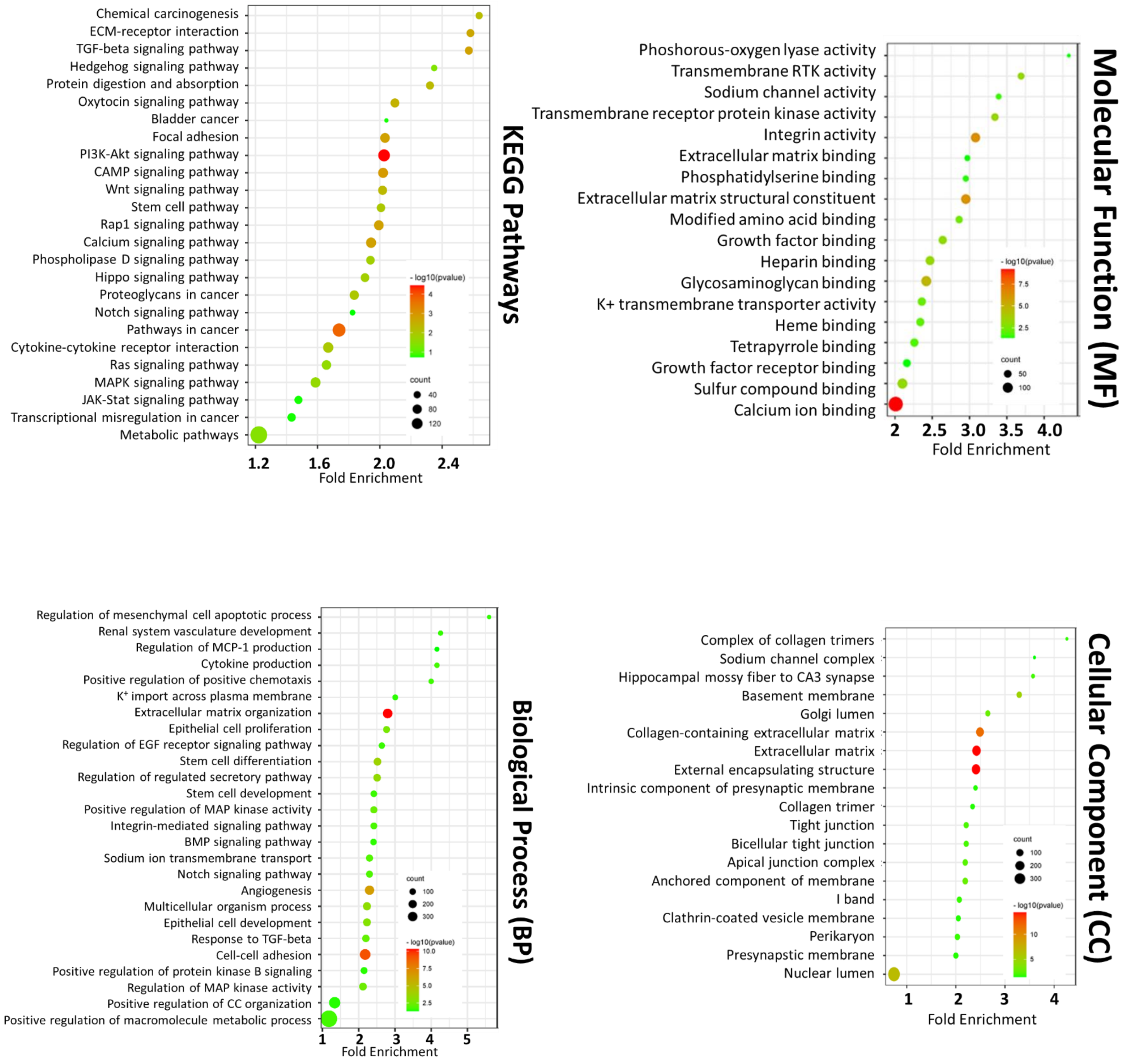

Supplementray Figure S4. Overall KEGG pathway and GO term analysis of differentially expressed genes based on RNA-seq data for 12 months As exposed cells compared to control cells. Each bubble's color and size correspond to the amount of differentially expressed mRNAs that are enriched in a certain GO keyword or KEGG pathway, respectively. The cut-off utilized to choose to GO and KEGG keywords were $p<0.05$.

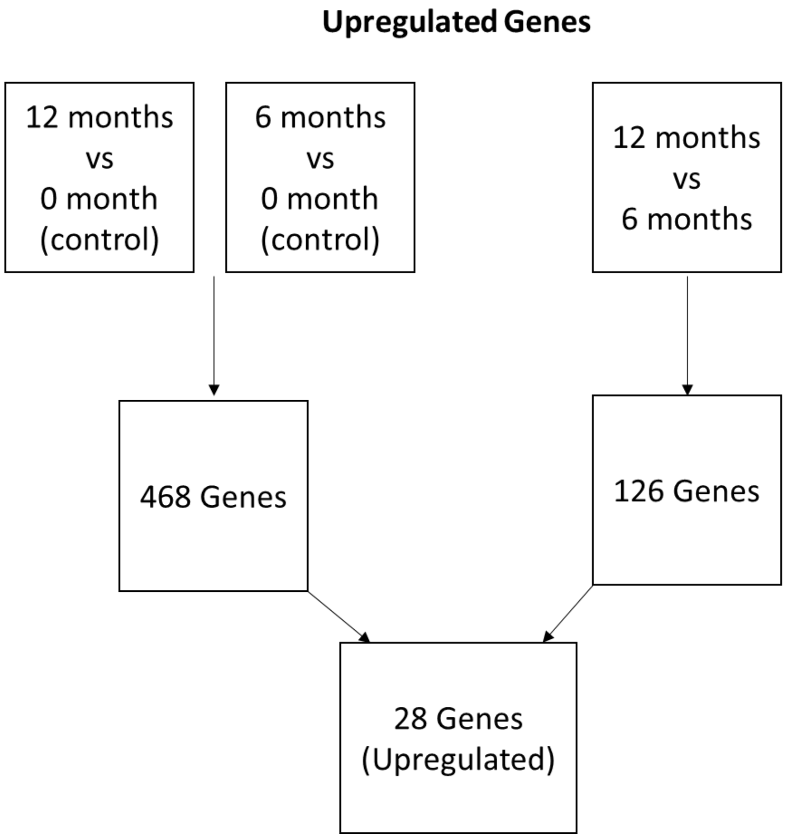

ALDH1A1, GUCY1A1, MUCL3, DAPK1, ATP10A, DSCAML1, ADAMTS10, RNF182, BCO1, PTH1R, SLC12A3, HLF, NPY1R, NPNT, SEC14L6, FABP6, ARHGAP6, GAL3ST1, MYL3, CP, WNT7A, HES7, STOX2, GCKR, RASAL1, DSCAML1, ADAMTS10, RNF182, BCO1, TMC5, TM4SF18, ZIC5

Supplementary Figure S5. Common upregulated genes between 12M vs 0M, 6M vs 0M and 12M vs 6M.

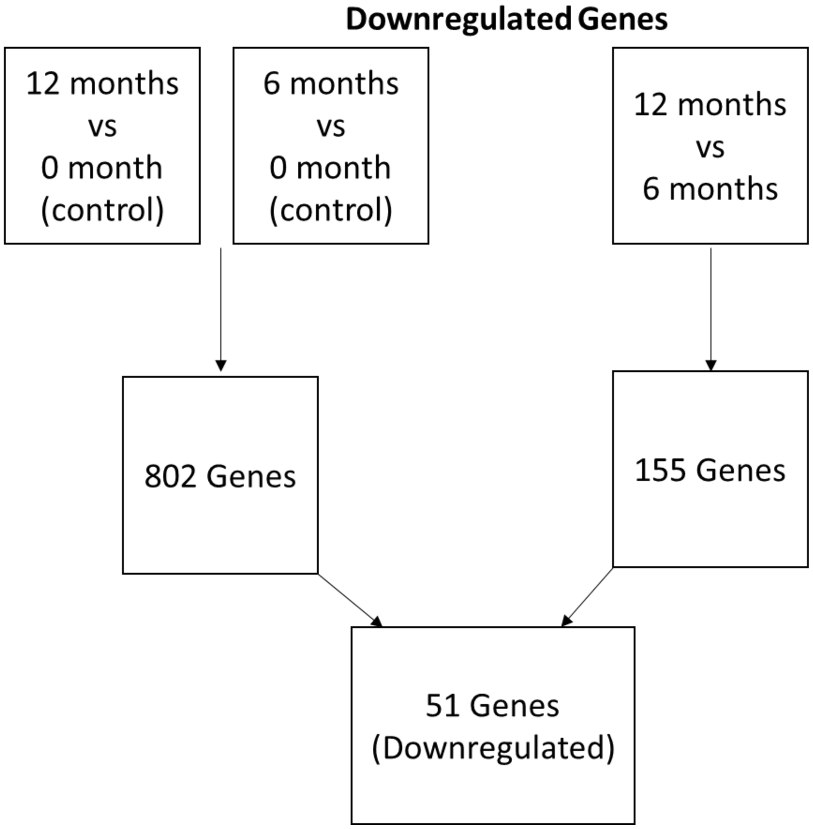

CPA4, SCG5, SHISAL1, MME, APCDD1L, SLC7A14, PNMA2, SLC18A3, BST2, DKK1, IGFBP4, TLR4, GALNT5, MEGF6, IL7R, ARNT2, GALC, MAP6, WLS, DHRS2, GOLGA7B, UNC5B, PLEKHG4B, DUSP2, DIO2, RTL5, ITGA11, IFI27, COL4A5, EPHB4, CDH11, ELOVL4 , FST, ANOS1, LAMA1, VGLL3, SLIT2, FAT4,TSPAN18, FAT3, VSTM2L, GABRA3, BGN, HTRA3, ACKR3, FMOD,PRXL2A, DCN, CCNA1, TRIB2, SPTBN5

Supplementary Figure S6. Common downregulated genes between 12M vs 0M, 6M vs 0M and 12M vs 6M.

Supplementary Table S1. List of primers used in the study

S.No.	Gene Name	Forward Primer	Reverse Primer	Amplicon Length
1	*ALDH1A1*	CTGCTGGCGACAATGGAGT	CGCAATGTTTTGATGCAGCCT	89
2	*HNF1B*	GTGGACCGGATGCTCAGTG	GGGTCTTCATAGGGGTGCC	160
3	*PTPRD*	TACAACCCTTACGGACTCCGA	GGAATTTGATCTTCCCGCAAAAC	115
4	*HAVCR1*	TGGCAGATTCTGTAGCTGGTT	AGAGAACATGAGCCTCTATTCCA	121
5	*PREX2*	TGGGAGGGGTCCAACATCA	TCTTCAACCGTCTGTGTTTTCTT	151
6	*PAX2*	TCAAGTCGAGTCTATCTGCATCC	CATGTCACGACCAGTCACAAC	92
7	*GAL3ST1*	CAAGACCCGGATCGCTACTAC	TGTCATAGCCCAGGTCGAAGA	85
8	*CDH1*	CGAGAGCTACACGTTCACGG	GGGTGTCGAGGGAAAAATAGG	119
9	*MAL*	TCACCTTGGACGCAGCCTA	GAAGCCGTCTTGCATCGTGAT	98
10	*NR1H4*	AAGAGATGGGAATGTTGGCT	TGCATGCTGCTTCACATTTT	98
11	*ACTIN*	GACGACATGGAGAAAATCTG	ATGATCTGGGTCATCTTCTC	131
12	*SULF1*	GATCCCCGAGGTTCAGAGGA	GGTGTAGTCACAAAGGCATTGA	178
13	*BEX1*	AAAGCGGACCAGCCCTTCT	CCTCCTTCTCCCGATTCTCG	81
14	*ITGA11*	GTGGCAATAAGTGGCTGGTC	GTTCCCGTGGATCACTGGAC	95
15	*COL4A5*	TGGACAGGATGGATTGCCAG	GGGGACCTCTTTCACCCTTAAAA	80
16	*CDH11*	AGAGGTCCAATGTGGGAACG	GGTTGTCCTTCGAGGATACTGT	109
17	*COL3A1*	GGAGCTGGCTACTTCTCGC	GGGAACATCCTCCTTCAACAG	78
18	*ANOS1*	TGGAAGTCGGAGCTCCCTT	CGGTTGACAGTGGGATCTTCT	85
19	*PTN*	GGAGCTGAGTGCAAGCAAAC	CTCGCTTCAGACTTCCAGTTC	157
20	*POSTN*	GACCGTGTGCTTACACAAATTG	AAGTGACCGTCTCTTCCAAG	128
21	*TPGS2*	GAGGACGATACACATGAAGCC	ACTGCCATTGCATGAATCCAG	96

Supplementary Table S2. Common and unique pathways between 12M vs 0M and 6M vs 0M

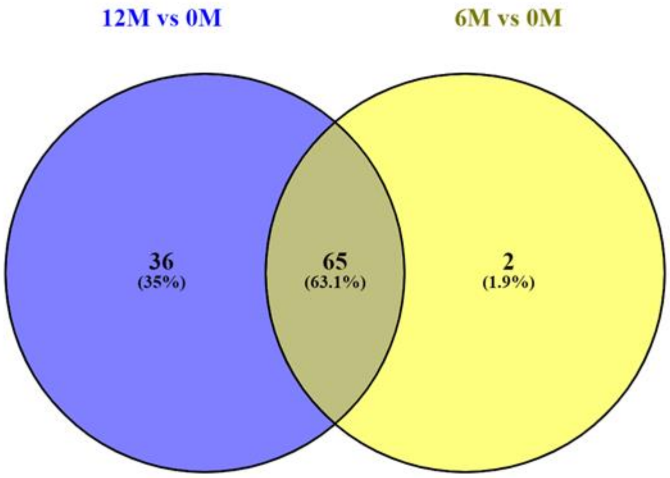

65 common elements in "12M vs 0M" and "6M vs 0M":	2 elements included exclusively in "6M vs 0M":	36 elements included exclusively in "12M vs 0M":
PI3K-Akt signaling pathway	Viral protein interaction with cytokine and cytokine receptor	Nitrogen metabolism
Human papillomavirus infection	Cardiac muscle contraction	Metabolic pathways
Pathways in cancer		Glycine, serine and threonine metabolism
Axon guidance		Inflammatory mediator regulation of TRP channels
Cell adhesion molecules		Alanine, aspartate and glutamate metabolism
ABC transporters		Viral myocarditis
Arrhythmogenic right ventricular cardiomyopathy		Steroid hormone biosynthesis
CAMP signaling pathway		Glycosaminoglycan biosynthesis
Calcium signaling pathway		Regulation of lipolysis in adipocytes
Focal adhesion		Maturity onset diabetes of the young

Complement and coagulation cascades		Tryptophan metabolism
Rap1 signaling pathway		Biosynthesis of amino acids
Neuroactive ligand-receptor interaction		Apelin signaling pathway
TGF-beta signaling pathway		Endocrine resistance
ECM-receptor interaction		Arachidonic acid metabolism
Dilated cardiomyopathy		Parathyroid hormone synthesis, secretion and action
Oxytocin signaling pathway		Endocytosis
Wnt signaling pathway		Epstein-Barr virus infection
Protein digestion and absorption		Ascorbate and aldarate metabolism
Circadian entrainment		Type II diabetes mellitus
Chemical carcinogenesis		Melanoma
Hypertrophic cardiomyopathy		Yersinia infection
AGE-RAGE signaling pathway in diabetic complications		Cortisol synthesis and secretion
Proteoglycans in cancer		Measles
Signaling pathways regulating pluripotency of stem cells		Inflammatory bowel disease
Cytokine-cytokine receptor interaction		Tight junction
Retinol metabolism		Primary bile acid biosynthesis
Phospholipase D signaling pathway		JAK-STAT signaling pathway
Hippo signaling pathway		Intestinal immune network for IgA production
Renin secretion		Dopaminergic synapse
Staphylococcus aureus infection		Taste transduction
Serotonergic synapse		Bladder cancer
Cushing syndrome		Graft-versus-host disease
Breast cancer		Pentose and glucuronate interconversions
MAPK signaling pathway		Drug metabolism
Ovarian steroidogenesis		Prostate cancer
Ras signaling pathway		
Platelet activation		
Fluid shear stress and atherosclerosis		
Basal cell carcinoma		
Cholinergic synapse		
Hepatitis C		
Hedgehog signaling pathway		
Leukocyte transendothelial migration		

Vascular smooth muscle contraction		
Hematopoietic cell lineage		
Regulation of actin cytoskeleton		
CGMP-PKG signaling pathway		
Gap junction		
Morphine addiction		
HIF-1 signaling pathway		
Small cell lung cancer		
Adrenergic signaling in cardiomyocytes		
Long-term depression		
Influenza A		
Purine metabolism		
Rheumatoid arthritis		
Amoebiasis		
Gastric acid secretion		
Leishmaniasis		
Malaria		
Coronavirus disease		
Transcriptional misregulation in cancer		
Glutamatergic synapse		
Notch signaling pathway		
